# Numerical material testing for discontinuous fiber composites using statistically similar representative volume elements

**DOI:** 10.1038/s41598-020-66963-1

**Published:** 2020-06-30

**Authors:** Takashi Sasagawa, Masato Tanaka, Ryuji Omote, Daniel Balzani

**Affiliations:** 1 Toyota Central R&D Laboratories, Inc., 41-1 Yokomichi, Nagakute, Aichi, 480-1192 Japan; 2 Institute of Mechanics and Shell Structures, Technical University Dresden, August-Bebel-Straße 30, 01219 Dresden, Germany; 3 Present Address: Chair of Continuum Mechanics, Ruhr-University-Bochum, Department of Civil- and Environmental Engineering, Universitätsstr. 150, 44801 Bochum, Germany

**Keywords:** Mechanical engineering, Computational methods, Nonlinear phenomena

## Abstract

A computational method is proposed in order to predict mechanical properties of discontinuous fiber composites (DFCs) based on computational homogenization with statistically similar representative volume elements (SSRVEs). The SSRVEs are obtained by reducing the complexity of real microstructures based on statistical measures. Specifically, they are constructed by minimizing an objective function defined in terms of differences between the power spectral density of target microstructures and that of the SSRVEs. In this paper, an extended construction method is proposed based on the reformulation of the objective function by integer design variables. The proposed method is applied to the representation of a real material, namely glass fiber reinforced nylon 6. The results show that the mechanical properties computed by numerical material tests using the SSRVEs agree with experimental results. Therefore, it is found that the nonlinear mechanical properties of the DFC can be suitably predicted by the proposed method without any special calibration to experiments performed on the composites.

## Introduction

Fiber reinforced plastics are advanced materials for lightweight design. Especially, discontinuous fiber composites (DFCs) are suited for mass production. DFCs making use of short fibers can be produced by injection molding, where a mixture of molten polymer and short fibers is injected into a mold. Therefore, flow directions depend on the position in the mold which has a significant effect on the microstructures of DFCs, e.g. the fiber orientations. As a result, the macroscopic properties of DFCs depend on the position in the product. In order to design a product made of DFCs to function well, it is necessary to know the mechanical properties of the material. However, it is hard to perform tests on material specimens taken from multiple positions in the product. For this reason, it is helpful to substitute the experimental tests by computational simulation of microstructures to predict the material properties.

There are two types of the methods for predicting material properties based on microstructures. One way is based on analytical approaches making use of the mean field theory such as Eshelby’s inclusion^1^, the Mori-Tanaka theory^2^ or the self-consistent model^3^. The other possibility is based on numerical approaches, such as numerical material testing^4–6^ making use of computational homogenization methods^7–11^. In analytical approaches, inclusions like fibers in microstructures are simplified with an ellipsoid and mechanical properties of composites are analytically derived based on micromechanics. Mechanical nonlinearity of the composite may be predicted by means of linearization of mechanical properties of matrix or inclusions in the incremental Eshelby-Mori-Tanaka procedures^12^. Fiber orientation distributions may also be considered with orientation tensors^13^ within the analytical methods^12^. In numerical approaches, representative volume elements (RVEs) are firstly constructed and then solved as microscopic boundary value problems using finite element method. Finally, macroscopic stress-strain relationships are obtained by volume averaging of microscopic stresses and strains. This so-called homogenization method is based on a separation of scales. The RVEs are usually portions of real microstructures or artificial microstructures which are faithful to real microstructures. Therefore, the method is able to consider not only fiber orientation distributions but also fiber length distributions, fiber spatial distributions and so on. In most cases, the periodic boundary condition is applied to the microscopic boundary value problems. This is due to its fast convergence rate to obtain macroscopic material properties with respect to size of RVE^14^. However, it can be difficult to apply the periodic boundary condition to RVEs for DFCs because their real microstructures are complex and nonperiodic. This leads to the necessity to consider relatively large RVEs and the high computational expenses. For example, Fliegener *et al*.^15^ have modeled RVEs of DFCs based on statistical measures obtained by analyzing computed tomography (CT) images and the resulting finite element model consisted of about 10 million elements.

Balzani *et al*.^16^ has been proposed statistically similar RVEs (SSRVEs). The SSRVEs are somewhat artificially generated such that two major goals are achieved: (i) a high statistical similarity regarding the microstructure morphology compared to the real microstructure data is ensured and (ii) a less complex morphology is obtained such that the periodic boundary condition can be applied resulting in reduced discretization costs. Only through these savings in computational effort, the fully scale-coupled simulation of materials taking into account microscopic simulations can be enabled. Furthermore, analysis in terms of virtual labs can be performed in significantly reduced time enabling a higher number of analysis and thus, a deeper understanding of the materials. For instance, the performances of the SSRVEs for dual-phase steels can be shown in the literatures^17–23^. In addition, the construction method of 2D statistical volume elements for unidirectional fiber composites has been proposed^24^. The original construction method of SSRVEs has also been applied to DFCs in the literatures^25,26^. The SSRVEs of DFCs consist of a large number of fibers oriented in different directions, which are thus quite different from the ones of dual-phase steels. For DFCs, the fibers in the SSRVEs can be modeled by cylinders of prescribed size and length distribution such that the SSRVEs can be constructed solely based on the power spectral density of target RVEs obtained from X-ray CT images. It was found that mechanical properties obtained by a series of numerical material tests using SSRVEs were similar to the target RVEs, but the computing time of numerical material tests for DFCs was significantly reduced. However, it is not clear that mechanical properties of real DFCs can be predicted using SSRVEs since so far only artificially generated target RVEs were analyzed. The difficulty in experimental validation of the SSRVEs is the spatial dispersion of mechanical properties of DFCs depending on the nonhomogeneity of their microstructure.

In this article, the calculation of mechanical properties of DFCs using SSRVEs is validated by comparing experimental results which are obtained from small specimens in order to avoid a significant influence of their spatial dispersion. Moreover, the optimization scheme with integer design variables is proposed in order to improve the efficiency of constructing the SSRVEs while only marginally compromising on the accuracy. This paper is organized as follows. Firstly, we provide an optimization scheme to construct SSRVEs for DFCs. Secondly, the new type of efficient optimization scheme using integer design variables are proposed and its performance through a simple example with a trivial solution is shown. Thirdly, we provide results of X-ray CT scans and experimental material tests of DFCs. Fourthly, SSRVEs are constructed using a real target microstructure obtained from X-ray CT images and our proposed method is validated by comparing the mechanical properties computed by numerical material tests using the SSRVEs with the experimental results. Finally, we summarize some concluding remarks.

## Method for the construction of SSRVEs

The SSRVEs are simplified RVEs with similar morphology to given target microstructures in the sense of statistics. Balzani *et al*.^16,17,27^ used the power spectral density (PSD), the lineal-path function and the Minkowski functionals as statistical descriptors for SSRVEs of a dual-phase steel. The periodicity of the size, shape, orientation and distance of inclusions can be well captured by the PSD as information of microstructures. In addition, the lineal-path function and the Minkowski functionals can capture the size and shape distribution of inclusions. Here, we assume that the microstructure morphology of DFCs can mostly be captured by the PSD since the size and shape distribution of inclusions are given by modeling fibers in the SSRVEs with cylinders whose length and diameter are prescribed. Investigations of the optimal statistical descriptors will be the subject of further studies. Then, the optimal parameterization $\tilde{{\boldsymbol{\gamma }}}$ of the SSRVE is obtained by the optimization problem

1 $$\tilde{{\boldsymbol{\gamma }}}={\rm{\arg }}\{\mathop{{\rm{\min }}}\limits_{{\boldsymbol{\gamma }}}[ {\mathcal E} ({\boldsymbol{\gamma }})]\},$$

wherein ${\mathcal E}$ is the objective function and ***γ*** is the parameterization of the microstructure morphology of the SSRVE which is defined as

2 $${\boldsymbol{\gamma }}={[{{\boldsymbol{\gamma }}}_{1}^{T},{{\boldsymbol{\gamma }}}_{2}^{T},\cdots ,{{\boldsymbol{\gamma }}}_{i}^{T},\cdots ,{{\boldsymbol{\gamma }}}_{{N}_{{\rm{fiber}}}}^{T}]}^{T}.$$

**Figure 1:**
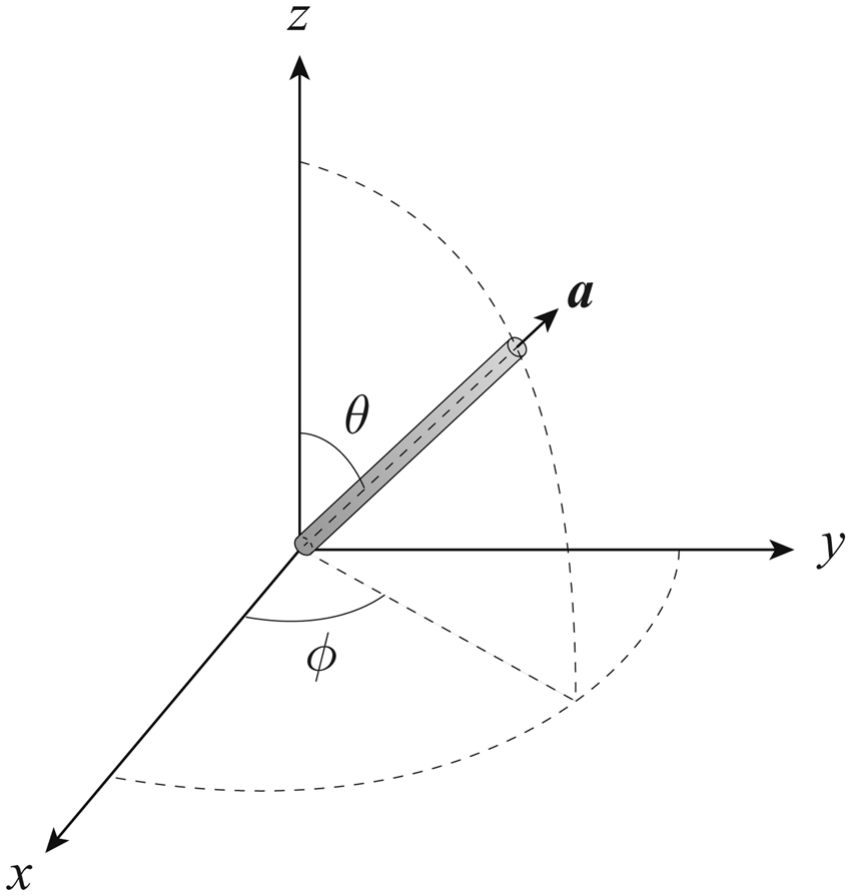
Definition of fiber angle in the longitudinal direction.

In the above expression, ***γ***_***i***_ is the design parameter vector for the *i*-th fiber of *N*_fiber_ fibers in the current SSRVE. The shape of the fibers is assumed to be cylindric in our model. The fiber orientation angles $(\phi ,\theta )$ depicted in Fig. 1 and the center coordinate values $({c}_{x},{c}_{y},{c}_{z})$ are stored in ***γ*** such that

3 $${{\boldsymbol{\gamma }}}_{i}=\{\begin{array}{ll}{\{{\phi }_{i},{\theta }_{i}\}}^{T}, & {\rm{if}}\,i=1,\\ {\{{c}_{x,i},{c}_{y,i},{c}_{z,i},{\phi }_{i},{\theta }_{i}\}}^{T}, & {\rm{otherwise}}.\end{array}$$

Here, note that only fiber orientation angles are stored in ***γ***_1_ since the center coordinates of the first fiber are fixated at the center of the SSRVE. Thereby, it is made sure that a simple translation of the inclusion phase is excluded which would neither modify the resulting homogenized response under periodic boundary conditions nor the statistical description, and which would thereby enable an infinite number of equal solutions to the optimization problem in (1). Furthermore, note that all parameters in ***γ***_***i***_ are assumed to be natural numbers if the integer-based method is to be applied. Thereby, only a subset of parameterizations in terms of whole numbers is enabled such that the unit of the physical dimensions of the parameter values defines the possible resolution of the parameterization. This restricts the flexibility of the optimization problem, it however enables a significantly more efficient optimization procedure. Note that for the implementation described in the next section we always consider the real-valued data type in the objective function, but an integer data type in the optimizer. This enables an easy switch between a real and integer optimization and it enables a more flexible modifiability of the parameterization. In this paper, the number, length, and diameter of the fibers and the size of the SSRVE are assumed to be constants reasonably defined based on measurements of the real microstructure.

In our optimization problem, the objective function ${\mathcal{E}}$ in (1) is written as

4 $$\begin{array}{ccc}\boldsymbol{\mathscr{E}}({\boldsymbol{\gamma }}) & = & \frac{1}{{\hat{N}}_{x}{\hat{N}}_{y}{\hat{N}}_{z}}\,\mathop{\sum }\limits_{j=1}^{{\hat{N}}_{x}}\,\mathop{\sum }\limits_{k=1}^{{\hat{N}}_{y}}\,\mathop{\sum }\limits_{l=1}^{{\hat{N}}_{z}}\,[{{\mathcal{S}}}_{jkl}({{\boldsymbol{\chi }}}^{{\rm{t}}{\rm{a}}{\rm{r}}{\rm{g}}{\rm{e}}{\rm{t}}},{R}_{x}^{{\rm{t}}{\rm{a}}{\rm{r}}{\rm{g}}{\rm{e}}{\rm{t}}},{R}_{y}^{{\rm{t}}{\rm{a}}{\rm{r}}{\rm{g}}{\rm{e}}{\rm{t}}},{R}_{z}^{{\rm{t}}{\rm{a}}{\rm{r}}{\rm{g}}{\rm{e}}{\rm{t}}})\\  &  & {-{{\mathcal{S}}}_{jkl}({{\boldsymbol{\chi }}}^{{\rm{S}}{\rm{S}}{\rm{R}}{\rm{V}}{\rm{E}}}({\boldsymbol{\gamma }}),{R}_{x}^{{\rm{S}}{\rm{S}}{\rm{R}}{\rm{V}}{\rm{E}}},{R}_{y}^{{\rm{S}}{\rm{S}}{\rm{R}}{\rm{V}}{\rm{E}}},{R}_{z}^{{\rm{S}}{\rm{S}}{\rm{R}}{\rm{V}}{\rm{E}}})]}^{2},\end{array}$$

where ${\mathcal{S}}$ is the rebinned PSD of the microstructure computed for a discrete set of ${\hat{N}}_{x}\times {\hat{N}}_{y}\times {\hat{N}}_{z}$ voxels. Note that subscripts “$x$”, “$y$” and “$z$” indicate the $x$-, $y$- and $z$-components and superscripts “target” and “SSRVE” indicate quantities of the target microstructure and the SSRVE, respectively. The voxel data ***χ*** is defined as

5 $${\chi }_{pqr}^{{\rm{A}}}=\{\begin{array}{cc}1, & {\rm{i}}{\rm{f}}\,{{\boldsymbol{x}}}_{pqr}^{{\rm{A}}}\in {D}^{{\rm{A}}},\\ 0, & {\rm{o}}{\rm{t}}{\rm{h}}{\rm{e}}{\rm{r}}{\rm{w}}{\rm{i}}{\rm{s}}{\rm{e}},\end{array}\,{\rm{w}}{\rm{i}}{\rm{t}}{\rm{h}}\,p=1,\cdots ,{N}_{x}^{{\rm{A}}},\,q=1,\cdots ,{N}_{y}^{{\rm{A}}},\,r=1,\cdots ,{N}_{z}^{{\rm{A}}},$$

which describes the binarized microstructure where ***x*** is the center coordinate vector of the corresponding voxels and $D$ is the domain of the fibers. Superscript “A” represents either “target” or “SSRVE”. In addition, ${N}_{x}$, ${N}_{y}$, ${N}_{z}$ indicate the size of ***χ*** and subscripts $p$, $q$, $r$ denote the indices of each voxel for ***χ*** in the $x$, $y$ and $z$ directions, respectively. Note that only ${D}^{{\rm{SSRVE}}}$ is a function of the design variables ***γ***.

The rebinned PSD ${\mathcal{S}}$ in (4) is given by

6 $${{\mathcal{S}}}_{jkl}({{\boldsymbol{\chi }}}^{{\rm{A}}},{R}_{x}^{{\rm{A}}},{R}_{y}^{{\rm{A}}},{R}_{z}^{{\rm{A}}})=\frac{{s}_{jkl}({{\boldsymbol{\chi }}}^{{\rm{A}}},{R}_{x}^{{\rm{A}}},{R}_{y}^{{\rm{A}}},{R}_{z}^{{\rm{A}}})}{{s}_{max}({{\boldsymbol{\chi }}}^{{\rm{A}}},{R}_{x}^{{\rm{A}}},{R}_{y}^{{\rm{A}}},{R}_{z}^{{\rm{A}}})},$$

7 $${s}_{jkl}({{\boldsymbol{\chi }}}^{{\rm{A}}},{R}_{x}^{{\rm{A}}},{R}_{y}^{{\rm{A}}},{R}_{z}^{{\rm{A}}})=\frac{1}{{R}_{x}^{{\rm{A}}}{R}_{y}^{{\rm{A}}}{R}_{z}^{{\rm{A}}}}\,{\sum }_{t={R}_{x}^{{\rm{A}}}(j-1)+1}^{j{R}_{x}^{{\rm{A}}}}\,{\sum }_{u={R}_{y}^{{\rm{A}}}(k-1)+1}^{k{R}_{y}^{{\rm{A}}}}\,{\sum }_{v={R}_{z}^{{\rm{A}}}(l-1)+1}^{l{R}_{z}^{{\rm{A}}}}\,{{\mathcal{P}}}_{tuv}({{\boldsymbol{\chi }}}^{{\rm{A}}}),$$

where ${s}_{{\rm{\max }}}$ is the maximum value of ***s*** and ${R}_{x}$, ${R}_{y}$, ${R}_{z}$ are the ratios between $\hat{N}$ and $N$ as shown by

8 $${R}_{x}^{{\rm{A}}}=\frac{{N}_{x}^{{\rm{A}}}}{{\hat{N}}_{x}},\,{R}_{y}^{{\rm{A}}}=\frac{{N}_{y}^{{\rm{A}}}}{{\hat{N}}_{y}},\,{R}_{z}^{{\rm{A}}}=\frac{{N}_{z}^{{\rm{A}}}}{{\hat{N}}_{z}}\mathrm{}.$$

Moreover, ${\mathcal{P}}$ in (7) is the PSD of the microstructure defined as

9 $${{\mathcal{P}}}_{tuv}({{\boldsymbol{\chi }}}^{{\rm{A}}})={|{{\mathcal{F}}}_{tuv}({{\boldsymbol{\chi }}}^{{\rm{A}}})|}^{2}={{\mathcal{F}}}_{tuv}^{\ast }({{\boldsymbol{\chi }}}^{{\rm{A}}}){{\mathcal{F}}}_{tuv}({{\boldsymbol{\chi }}}^{{\rm{A}}}),$$

where ${\mathcal F}$ is the ${N}_{x}\times {N}_{y}\times {N}_{z}$ voxel data obtained from the Fourier transform of ${\boldsymbol{\chi }}$ and ${ {\mathcal F} }^{\ast }$ is the conjugate complex of ${\mathcal F}$.

It is worth noting that the total number ${N}^{{\rm{target}}}$ of voxels of ${{\boldsymbol{\chi }}}^{{\rm{target}}}$ can be different from the total number ${N}^{{\rm{SSRVE}}}$ of voxels of ${{\boldsymbol{\chi }}}^{{\rm{SSRVE}}}$. This will automatically be the case when the number of voxels in the physical space, i.e. of the microstructure morphology itself, differs because the target microstructure is larger than the SSRVE. This can be easily avoided by rebinning the PSD of the SSRVE and the target microstructure as shown in (7) and (8). Before the calculation of the rebinned PSD ${\mathcal{S}}$, we remove the trivial entry ${{\mathcal{P}}}_{111}$ since it provides no information and is just redundant. Furthermore, note that the fibers sticking out of the SSRVE are cut on the surface of the SSRVE and they are transferred into the SSRVE such that the fiber distribution is periodic in the construction of ${\chi }_{pqr}^{{\rm{A}}}$.

The objective function ${\mathcal E}$ in (1) is discrete, non-differentiable and generally non-convex^16^. Therefore, we use the global optimization toolbox in MATLAB (R2016b) which is based on genetic algorithms. In addition to that, we use the fast Fourier transform in MATLAB for computation of ${\mathcal F}$ in (9). Note that a global minimum might not be guaranteed due to the non-convexity of the objective function ${\mathcal E}$. However, it has been shown that the obtained minimum is considered appropriate in the sense that the mechanical response of the SSRVE is similar to the target microstructure^25,26^. In this paper, the obtained minimum will be also validated in an additional sense that the mechanical response of the SSRVE is similar not only to that of the target microstructure but also to that of the experimental results.

## Cost reduction using integer optimization

This section provides details regarding the optimization scheme using integer design variables, which is called integer optimization. Additionally, it is verified in a simple example by comparing its efficiency to the optimization scheme using design variables of real-valued data type.

### Algorithmic treatment using integer optimization

The morphology of the SSRVE is discretized with the voxel data ***χ*** which is a function of ***γ*** as shown in (5). In this paper, ***γ*** is also discretized in order to reduce the computational cost of the optimization scheme, thereby however restricting the flexibility of the parameterization. The fiber center coordinates $({c}_{x},\,{c}_{y},\,{c}_{z})$ and the fiber orientation angles $(\phi ,\theta )$ are implemented as integer variables by following the steps below:

1. Define the design variables ${c}_{x}^{{\rm{integer}}},{c}_{y}^{{\rm{integer}}},{c}_{z}^{{\rm{integer}}},{\phi }^{{\rm{integer}}},{\theta }^{{\rm{integer}}}$ as integer variables.
2. Define a set of lower and upper bounds on the design variables as
  10 $$0\le {c}_{x,i}^{{\rm{integer}}}\le {N}_{x}^{{\rm{SSRVE}}},\,0\le {c}_{y,i}^{{\rm{integer}}}\le {N}_{y}^{{\rm{SSRVE}}},\,0\le {c}_{z,i}^{{\rm{integer}}}\le {N}_{z}^{{\rm{SSRVE}}},$$
  11 $$0\le {\phi }_{i}^{{\rm{integer}}}\le Int\left[\frac{\pi }{\Delta {\varphi }_{i}}\right],\,0\le {\theta }_{i}^{{\rm{integer}}}\le Int\left[\frac{\pi }{\Delta {\varphi }_{i}}\right],$$
  with the operator Int[·] to convert a real variable into an integer variable and the minimum angle Δ${\varphi }_{i}$ defined as
  12 $$\Delta {\varphi }_{i}=\arctan \left(\frac{\Delta x}{{L}_{i}^{{\rm{fiber}}}}\right),$$
  with the voxel size Δ*x* of ${\boldsymbol{\chi }}$ and the length ${L}_{i}^{{\rm{fiber}}}$ of the $i$-th fiber.
3. Call a solver for nonconvex global optimization (e.g. in our case the genetic algorithm solver in the global optimization toolbox of MATLAB) with the set of the bounds and the option for the integer optimization.
4. Convert the integer design variables back into the following real-valued design variables in the subroutine computing the objective function
  13 $${c}_{x,i}^{{\rm{real}}}={\rm{Real}}[{c}_{x,i}^{{\rm{integer}}}]\Delta x,\,{c}_{y,i}^{{\rm{real}}}={\rm{Real}}[{c}_{y,i}^{{\rm{integer}}}]\Delta x,\,{c}_{z,i}^{{\rm{real}}}={\rm{Real}}[{c}_{z,i}^{{\rm{integer}}}]\Delta x,$$
  14 $${\phi }_{i}^{{\rm{real}}}={\rm{Real}}[{\phi }_{i}^{{\rm{integer}}}]\Delta {\varphi }_{i},\,{\theta }_{i}^{{\rm{real}}}={\rm{Real}}[{\theta }_{i}^{{\rm{integer}}}]\Delta {\varphi }_{i},$$
  with the operator Real[·] to convert an integer variable into a real variable.
5. Compute the objective function with the real-valued design variables.
6. Update the integer design variables with the genetic algorithms solver and return to 4.

Note that in principle an optimized implementation could be obtained by also implementing the objective function in terms of integer variables. However, this would restrict the simple extensibility in terms of advanced parameterizations. Moreover, the gain in efficiency by using integers in the optimization process is orders of magnitude higher than the gain in computing time for the single evaluation of the objective function by using integer data type within the objective function.

### Comparison between integer and real optimizations

**Figure 2:**
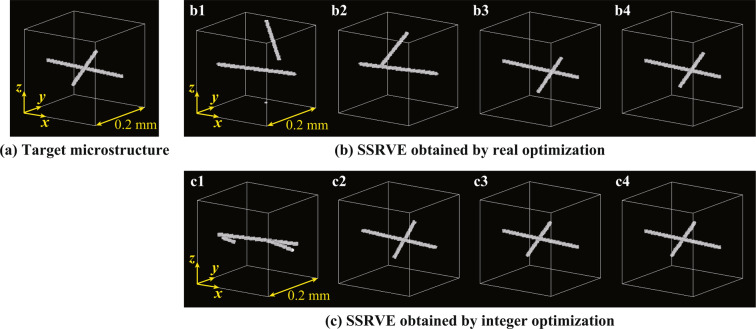
Fiber distributions of target microstructure and SSRVEs for the first (**b1**,**c1**), 10th (**b2**,**c2**), 50th (**b3**,**c3**), and 100th (**b4**,**c4**) generation in real and integer optimizations. The images have depicted using the 3D image processing software Volume Extractor 3.0.

**Figure 3:**
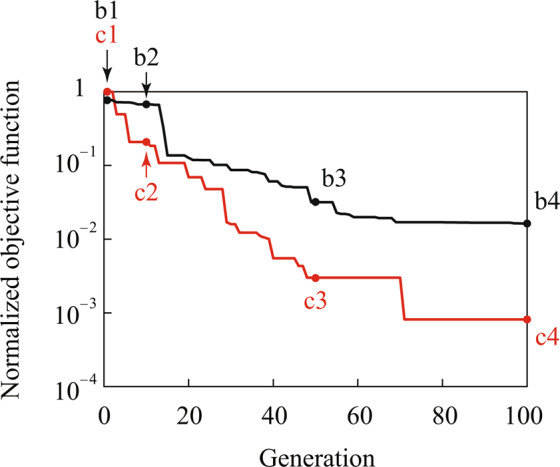
Convergence behavior of objective functions in real optimization (black line) and integer optimization (red line). The vertical axis represents the minimum value of objective function out of the populations normalized with respect to the first generation in the integer optimization. The symbols b1–b4 and c1–c4 show the objective functions for the first, 10th, 50th and 100th generation in real and integer optimizations, respectively.

The advantage of the integer optimization is shown by comparing the proposed approach to an optimization scheme with real-valued design variables, which is called real optimization. In the real optimization, a set of lower and upper bounds on the real-valued design variables are defined as

15 $$0\le {c}_{x,i}^{{\rm{real}}}\le {L}_{x}^{{\rm{SSRVE}}},\,0\le {c}_{y,i}^{{\rm{real}}}\le {L}_{y}^{{\rm{SSRVE}}},\,0\le {c}_{z,i}^{{\rm{real}}}\le {L}_{z}^{{\rm{SSRVE}}},\,0\le {\phi }_{i}^{{\rm{real}}}\le \pi ,\,0\le {\theta }_{i}^{{\rm{real}}}\le \pi ,$$

with the size $({L}_{x}^{{\rm{SSRVE}}},{L}_{y}^{{\rm{SSRVE}}},{L}_{z}^{{\rm{SSRVE}}})$ of the SSRVE in the $x$, $y$ and $z$ directions instead of (10) and (11). The simplified target microstructure considered here consists of two fibers embedded in a matrix as shown in Fig. 2(a). The diameter of the two fibers is 8.0 μm and the length is 0.2 mm and 0.1 mm, respectively. The SSRVEs also consist of two fibers whose diameter and length equal the ones in the target microstructure. Therefore, the morphology of the SSRVEs is expected to be identical to that of the target microstructure by solving the optimization problem. The morphology of the SSRVEs changes during the real and integer optimization processes as shown in Fig. 2(b,c), respectively. As a result, the objective functions decrease during the optimizations as depicted in Fig. 3. The symbols (b1)–(b4) and (c1)–(c4) in Figs. 2 and 3 indicate the SSRVEs and the objective functions for the first, 10th, 50th and 100th generations in the optimizations, respectively. The parameters of the optimization scheme in this example are summarized in Table 1. For the genetic algorithms, the population size was set to 60 and the optimization iteration stopped when the number of generations reaches 100. All other parameters in the global optimization toolbox were set to default values in MATLAB. Additionally, shared memory parallel procedure was applied using the Parallel Computing Toolbox in MATLAB with an Intel Core Processor (Broadwell) 2.6 GHz (16 Processors) with a NVIDIA Quadro M4000 GPU and distributed memory parallel computing will be the subject of further studies.

**Table 1:** Parameters of optimization scheme.

${\hat{{\boldsymbol{N}}}}_{{\boldsymbol{x}}}$	${\hat{{\boldsymbol{N}}}}_{{\boldsymbol{y}}}$	${\hat{{\boldsymbol{N}}}}_{{\boldsymbol{z}}}$	${{\boldsymbol{N}}}_{{\boldsymbol{x}}}^{{\bf{target}}}$	${{\boldsymbol{N}}}_{{\boldsymbol{y}}}^{{\bf{t}}{\bf{a}}{\bf{r}}{\bf{g}}{\bf{e}}{\bf{t}}}$	${{\boldsymbol{N}}}_{{\boldsymbol{z}}}^{{\bf{t}}{\bf{a}}{\bf{r}}{\bf{g}}{\bf{e}}{\bf{t}}}$	${{\boldsymbol{N}}}_{{\boldsymbol{x}}}^{{\bf{SSRVE}}}$	${{\boldsymbol{N}}}_{{\boldsymbol{y}}}^{{\bf{S}}{\bf{S}}{\bf{R}}{\bf{V}}{\bf{E}}}$	${{\boldsymbol{N}}}_{{\boldsymbol{z}}}^{{\bf{SSRVE}}}$	Δ*x*
75	75	75	75	75	75	75	75	75	2.67 μm

The SSRVE for the 100th generation in the integer optimization is identical to the target microstructure. In contrast, the shorter fiber in the SSRVE for the 100th generation in the real optimization is off to the right, that is, the SSRVE obtained by the real optimization is similar but still different to the target microstructure. Moreover, the objective function in the integer optimization converges to zero more rapidly than the real optimization, see Fig. 3. For the real optimization it has to be expected that the final optimum was not yet found within 100 generations. Therefore, it is found that the integer optimization scheme works more efficiently in the present example.

## Materials and Experiments

This section provides results of X-ray CT scans and material tests of DFCs. Their results are used for construction and validation of SSRVEs.

### Specimens and X-ray CT scans

The considered test pieces are cubes with a length of 2 mm cut out of a $400\times 400\times 2\,{{\rm{mm}}}^{3}$ plate produced by injection molding using glass fiber reinforced nylon 6 pellets (1015GNKF) provided by Ube Industries, Ltd.. The fiber weight fraction in the pellets is 30% which can be translated into approximately 16% of fiber volume fraction with the density 1.13 g/cm^3^ of nylon 6 and the density 2.54 g/cm^3^ of glass fiber. The injection molding machine (MD450S-IV) developed by Ube Machinery corporation, Ltd. was used and the molding conditions were set as in Table 2. Several specimens whose microstructures are similar to each other are needed for evaluation of anisotropic properties because a mechanical property in only one direction can be measured from one specimen. Therefore, the test pieces were cut out from the region where the flow direction did not really change in a simulation using the injection molding CAE software, 3D TIMON.

**Table 2:** Injection molding conditions.

Melt temperature	280 °C
Mold temperature	60 °C
Clamping pressure	35 MPa
Injection time	1.53 sec
Holding time	5 sec
Cooling time	60 sec

**Figure 4:**
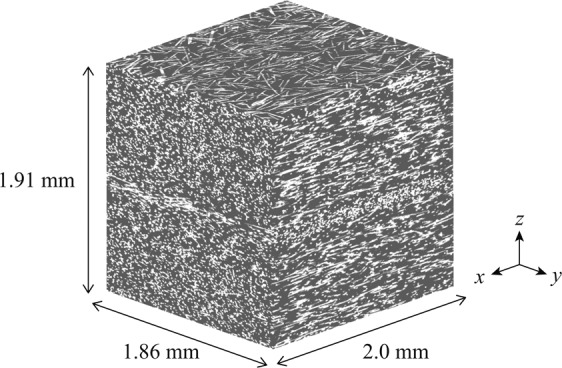
Microstructure of DFCs measured by X-ray CT. The image has depicted using the 3D image processing software Simpleware 2016.09.

X-ray CT scanning was performed using the three dimensional measuring X-ray CT Scanner (TDM1000H-II) developed by Yamato Scientific Co., Ltd. and parameters of the X-ray CT scanning are shown in Table 3. The microstructure of the DFCs was obtained by reconstruction of X-ray CT images shown in Fig. 4. Furthermore, a fiber orientation tensor was computed by the image analysis using the high-end industrial CT software VGSTUDIO MAX 2.2 based on the structure tensor approach28. Fiber orientation tensors obtained from all specimens are similar each other and the representative orientation tensor is shown as

16 $${\boldsymbol{A}}=(\begin{array}{ccc}0.620 & 0.003 & 0.0002\\  & 0.235 & 0.0005\\ sym\mathrm{}. &  & 0.145\end{array})\mathrm{}.$$

**Table 3:** Parameters of X-ray CT measurements.

Voxel size	5.74 μm
Voltage	45 kV
Current	15 μA
Number of frames per view	20
Number of views per 360 degrees	1200

The second order orientation tensor^13^ is defined as

17 $${A}_{ij}=\int \,{a}_{i}{a}_{j}\psi ({\boldsymbol{a}})d\,\,\,\,\,{\rm{w}}{\rm{i}}{\rm{t}}{\rm{h}}\,\,\,{a}_{1}=\sin \theta \cos \phi ,\,{a}_{2}=\sin \theta \sin \phi ,\,{a}_{3}=\cos \theta ,$$

where ***a*** is the unit vector describing the longitudinal direction of a fiber as shown in Fig. 1 and $\psi ({\boldsymbol{a}})$ is the probability density function for the fiber orientation ***a***. The corresponding diagonal component ${A}_{11}$, ${A}_{22}$ or ${A}_{33}$ becomes larger as fibers are more strongly oriented in the $x$, $y$ or $z$ direction. The obtained orientation tensor shows that most of the fibers in the test piece are oriented in the $x$ direction, which is the flow direction in the injection molding process, since ${A}_{11}$ is larger than ${A}_{22}$ and ${A}_{33}$.

### Material tests

**Figure 5:**
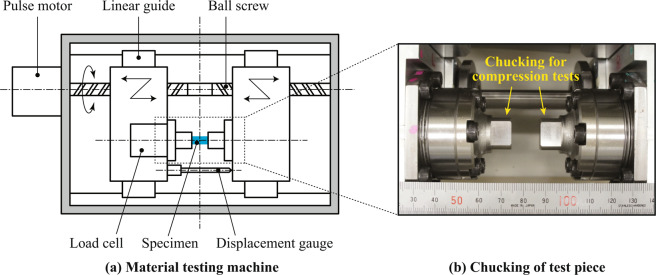
Experimental setup for material testing.

**Figure 6:**
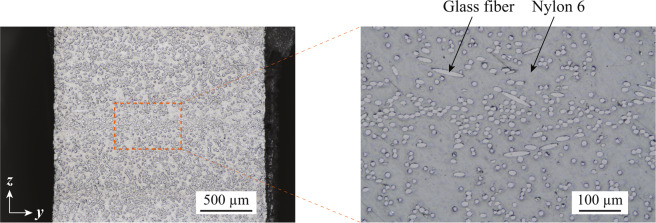
Polished surfaces of test pieces for compression tests.

Compression tests were conducted at room temperature using the material testing machine shown in Fig. 5 and the test pieces after the X-ray CT scans. The surfaces of the test pieces were polished as shown in Fig. 6 using the MultiPrep™ polishing system and aluminum oxide lapping films (9 to 3 micron) produced by Allied High Tech Products, Inc. in order to reduce stress concentrations caused by their unevenness. The specimens were dried in a vacuum oven for 12 hours at 80 °C before the testing in order to exclude the influence of water absorption on the mechanical properties. The test pieces were chucked by steel blocks shown in Fig. 5 and compressed from both sides at 10 μm per minute. Note that fluorinated grease was applied to the edges of the specimens to reduce friction between the test pieces and the steel blocks.

**Figure 7:**
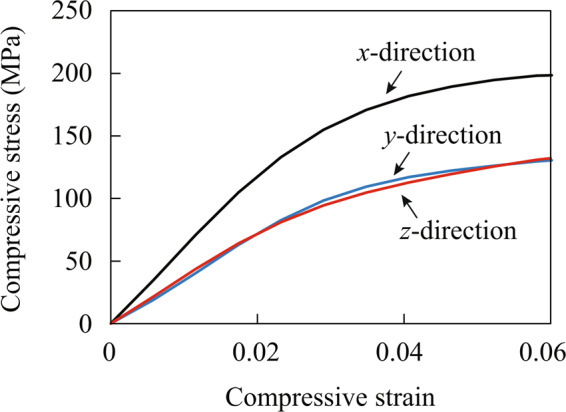
Nominal stress versus nominal strain curves obtained by compression tests in $x$, $y$ and $z$ directions.

The mechanical properties obtained by the compression tests in the $x$, $y$ and $z$ directions are summarized in Fig. 7. The nominal stress and strain were computed by dividing loading and stroke by area and length of the samples, respectively. Figure 7 shows that the stiffness in the $x$ direction where the fibers are mostly oriented is larger than the other directions and the mechanical properties are nonlinear in all directions.

## Computational analysis

Here, SSRVEs are constructed using the target microstructure obtained by X-ray CT in the previous section. Then, the SSRVEs are validated by comparing the mechanical properties obtained by numerical material tests using the SSRVEs with the experimental results and analytical results based on the Mori-tanaka theory.

### Construction of SSRVEs

The voxel data ${{\boldsymbol{\chi }}}^{{\rm{t}}{\rm{a}}{\rm{r}}{\rm{g}}{\rm{e}}{\rm{t}}}$ describing the target microstructure in (5) was obtained by binarizing the X-ray CT images. The fiber volume fraction of the target microstructure is approximately 16% as mentioned in the previous section. The mean fiber diameter and the mean fiber length are approximately 0.014 mm and 0.3 mm, respectively, which were measured using optical microscope images of fibers obtained by burning out the polymer matrix. Therefore, the diameter and the length of fibers in the SSRVEs were set to 0.014 mm and 0.3 mm, respectively. Then, the number of fibers and the dimensions of the SSRVEs were set to 30 and 0.207 × 0.207 × 0.207 mm^3^, respectively, such that the fiber volume fraction of the SSRVEs is as similar as possible to that of the target microstructure. The orientation and spatial distribution of the fibers in the SSRVE were computed by solving the optimization problem given in (1). The parameters of the optimization scheme in this analysis are summarized in Table 4. For the genetic algorithms, the population size was set to 200 and the optimization iteration stopped when the number of generations reached 300. Any other parameters were set to default values in the global optimization toolbox in MATLAB.

**Table 4:** Parameters of optimization scheme.

${\hat{{\boldsymbol{N}}}}_{{\boldsymbol{x}}}$	${\hat{{\boldsymbol{N}}}}_{{\boldsymbol{y}}}$	${\hat{{\boldsymbol{N}}}}_{{\boldsymbol{z}}}$	${{\boldsymbol{N}}}_{{\boldsymbol{x}}}^{{\bf{target}}}$	${{\boldsymbol{N}}}_{{\boldsymbol{y}}}^{{\bf{target}}}$	${{\boldsymbol{N}}}_{{\boldsymbol{z}}}^{{\bf{target}}}$	${{\boldsymbol{N}}}_{{\boldsymbol{x}}}^{{\bf{SSRVE}}}$	${{\boldsymbol{N}}}_{{\boldsymbol{y}}}^{{\bf{SSRVE}}}$	${{\boldsymbol{N}}}_{{\boldsymbol{z}}}^{{\bf{SSRVE}}}$	Δ*x*
12	12	12	312	312	312	36	36	36	5.74 μm

**Figure 8:**
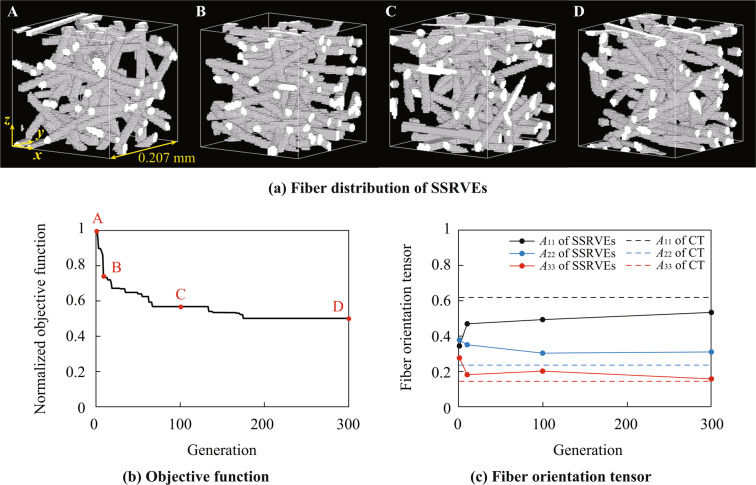
(**a**) SSRVEs constructed from design variables at the the first (A), 10th (B), 100th (C) and 300th (D) generations in optimization scheme. (**b**) Convergence behavior of objective function during optimization using the genetic algorithm. The vertical axis shows the minimum value of objective function out of the populations normalized with respect to the first generation. (**c**) Changes of components of fiber orientation tensor computed from design variables during optimization. Circles and dashed lines show components of fiber orientation tensor computed from SSRVEs and CT images, respectively. The images in (**a**) have depicted using the 3D image processing software Volume Extractor 3.0.

The SSRVEs was obtained by the optimization as shown in Fig. 8(a) where only the fiber domain satisfying ${\chi }_{pqr}^{{\rm{S}}{\rm{S}}{\rm{R}}{\rm{V}}{\rm{E}}}=1$ is displayed. The objective function, which was again defined in terms of the difference between the PSD of the target microstructure and that of the SSRVEs, decreased during the optimization as depicted in Fig. 8(b). Therefore, it was found that the proposed optimization scheme works well in the present analysis. The fiber orientation tensors were computed from design variables at first, 10th, 100th and 300th generations in optimization scheme as shown in Fig. 8(c). The fiber orientation tensor of the SSRVEs asymptotically approached the one obtained from the CT images. Note that the influence of the changes in morphology shown in Fig. 8(a) on the accuracy of the mechanical properties are summarized in the next section.

### Model description for numerical material tests

The SSRVEs in Fig. 8(a) have been discretized by voxel-based meshing using the 3D image processing software Simpleware 2016.09. Note that $72\times 72\times 72$ voxels describing the morphology of each SSRVE have been used and then each voxel has been discretized by one hexahedral element. As a result, the finite element models of the SSRVEs consist of 389017 nodes and 373248 hexahedral elements with reduced integration (C3D8R) in the implicit finite element commercial software Abaqus/Standard version 6.14. Note that the fiber-matrix interface is completely bonded because the interfacial shear strength of glass fiber reinforced polyamide 6 can be higher than the strength of the matrix^28^. Moreover, viscoelastic and fracture modelling of the glass fiber and polyamide 6 will be the subject of further studies.

Fibers and matrix in the SSRVEs have been modeled using an elasto-plastic model where geometrical nonlinearity is considered. The Jaumann rate of the Kirchhoff stress

18 $${{\boldsymbol{\tau }}}^{{\rm{\nabla }}}=\dot{{\boldsymbol{\tau }}}-{\boldsymbol{W}}{\boldsymbol{\tau }}+{\boldsymbol{\tau }}{\boldsymbol{W}}={\mathbb{C}}:({\boldsymbol{D}}-{{\boldsymbol{D}}}^{p}),$$

is used for the model, where ${\boldsymbol{\tau }}$ is the Kirchhoff stress, the superposed dot indicates the material time derivative, ${\mathbb{C}}$ is the tangent modulus tensor, ***D*** is the symmetric parts of the spatial velocity gradient and ***W*** is the antisymmetric parts of the one, respectively. Herein, ***D*** is decomposed into the elastic part and the plastic part as

19 $${\boldsymbol{D}}={{\boldsymbol{D}}}^{e}+{{\boldsymbol{D}}}^{p}\mathrm{}.$$

***D***^*p*^ follows the associated flow rule

20 $${{\boldsymbol{D}}}^{p}=\gamma \frac{\partial f}{\partial {\boldsymbol{\sigma }}}=\gamma \frac{{\boldsymbol{\sigma }}}{\Vert {\boldsymbol{\sigma }}\Vert },$$

where *γ* and ***σ*** are the plastic multiplier and the Cauchy stress, respectively. In addition, *f* is the von Mises yield criterion defined as

21 $$f({\boldsymbol{\sigma }},\alpha )=\Vert {{\boldsymbol{\sigma }}}^{{\rm{dev}}}\Vert -\sqrt{\frac{2}{3}}\{{\sigma }_{y}+H(\alpha )\alpha \}=\sqrt{{{\boldsymbol{\sigma }}}^{{\rm{dev}}}:{{\boldsymbol{\sigma }}}^{{\rm{dev}}}}-\sqrt{\frac{2}{3}}\{{\sigma }_{y}+H(\alpha )\alpha \},$$

with the equivalent plastic strain $\alpha$, the initial yield stress ${\sigma }_{y}$ and the isotropic hardening coefficient $H$. Additionally, ***σ***^dev^ is the deviatoric component of Cauchy stress formulated as

22 $${{\boldsymbol{\sigma }}}^{{\rm{dev}}}={\boldsymbol{\sigma }}-\frac{{\rm{tr}}({\boldsymbol{\sigma }})}{3}{\boldsymbol{I}},$$

with the trace tr(***σ***) of ***σ*** and the 2nd-order identity tensor ***I***. Furthermore, $\alpha$ in (21) follows the evolution equation

23 $$\dot{\alpha }=\sqrt{\frac{2}{3}}\Vert {{\boldsymbol{D}}}^{p}\Vert =\gamma \sqrt{\frac{2}{3}}.$$

**Figure 9:**
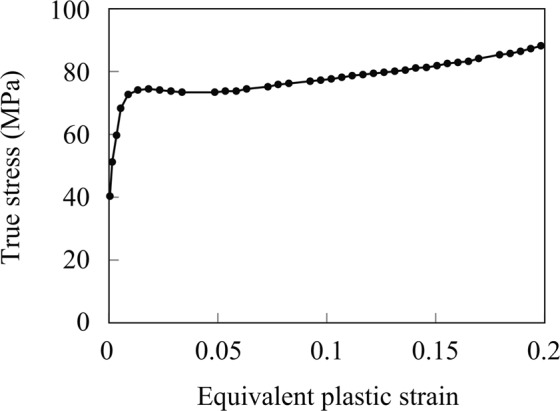
Hardening behavior of polyamide 6 based on the literature^30^. The stress-strain data at the circles were inputed in Abaqus/Standard.

The material parameters for the fibers and the matrix have been identified based on the material properties of the glass fiber (E-glass)^29^ and polyamide 6^30^ as shown in Table 5. The isotropic hardening coefficient $H$ was set in a way that it matches with the real hardening behavior of polyamide 6 shown in Fig. 9 using *PLASTIC in Abaqus/Standard.

**Table 5:** Material parameters of fibers and matrix for numerical material testing.

	Fiber	Matrix
Young’s modulus (GPa)	74	2.78
Poisson’s ratio	0.2	0.35
Yield stress *σ*_*y*_ (MPa)	—	27.8

Virtual compression tests in the $x$, $y$ and $z$ directions have been performed under periodic boundary conditions. The periodic boundary conditions were implemented by using multi-point constraint equations in Abaqus/Standard, cf. the literature^4^. The compression analyses were employed using an Intel Xeon processor E5-2667 v2 with the NVIDIA Tesla K20X GPUs.

### Numerical material tests

**Figure 10:**
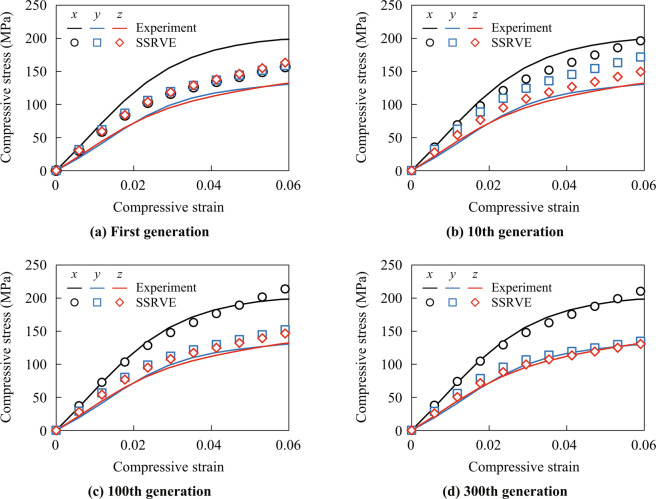
Comparison between experimental results and numerical results using SSRVEs for the first, 10th, 100th and 300th generation during the optimization procedure. Black, blue and red lines show the stress-strain curves obtained by the material tests in the $x$, $y$ and $z$ directions, respectively. Circles, squares and rhombuses indicate the stress-strain curves obtained by the numerical material tests in the $x$, $y$ and $z$ directions, respectively.

The relationships between compressive stress and strain were obtained by numerical material tests using the SSRVEs obtained for the first, 10th, 100th and 300th generation in the optimization scheme as shown in Fig. 10. The homogenized compressive stress and strain are given as volume averages of the nominal stress and strain of the elements in the SSRVEs, respectively. They were computed using the reaction forces and the displacements of control nodes, cf. the literature^4^. The compressive stress and strain in Fig. 10 represent their components in the loading direction.

The stress and strain curves for the first generation are more or less the same for all loading directions because the fiber orientation distribution in the associated SSRVE has been purely determined by random values. While the morphology of the SSRVEs is improved during the optimization process, their mechanical properties approach the anisotropic experimental results more and more, such that after the 300th generation the numerical stress-strain response agrees well with the experiments. Therefore, it was found that the nonlinear mechanical properties of the DFC could be predicted using an SSRVE based on the power spectral density of the real microstructure in this example.

### Comparison with analytical results

In order to compare the accuracy of the proposed method with an analytical homogenization technique, mechanical properties of the DFC were calculated using the commercial micromechanical material modeling software Digimat-MF based on the Mori-tanaka theory. The *J*_2_-plasticity model with the following yield criterion has been applied:

24 $$f({\boldsymbol{\sigma }},\alpha )=\sqrt{{{\boldsymbol{\sigma }}}^{{\rm{dev}}}:{{\boldsymbol{\sigma }}}^{{\rm{dev}}}}-\sqrt{\frac{2}{3}}\{{\sigma }_{y}+K\alpha +{R}_{inf}[1-\exp (-M\alpha )]\},$$

with the linear hardening coefficient *K*, the hardening modulus *R*_inf_ and the hardening exponent *M*. The Young’s moduli, Poisson’s ratios, and yield stresses for the fibers and the matrix are identical to the ones for the numerical material tests as shown in Table 5. $K$, ${R}_{inf}$ and $M$ were set to 88.4 MPa, 41.9 MPa and 503, respectively, such that they match with the real hardening behavior of polyamide 6 shown in Fig. 9 as much as possible using the nonlinear curve-fitting function, lsqcurvefit, in MATLAB. The fiber volume fraction, fiber diameter and fiber length are set to 16%, 0.014 mm and 0.3 mm, respectively.

**Figure 11:**
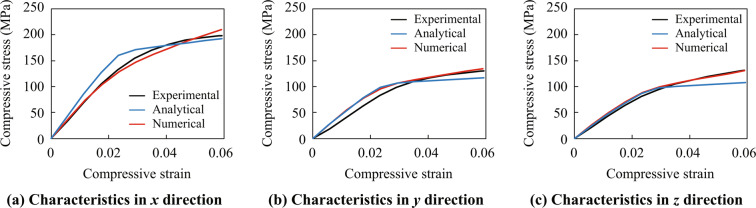
Comparison between analytical results based on Mori-Tanaka theory, numerical results using SSRVEs, and experimental results.

The analytical results were obtained as shown in Fig. 11. As expected, the results show that the numerical results, which are computed by the proposed methods, agree with experimental results better than the analytical results.

## Conclusion

A computational method was proposed in order to predict the mechanical properties of DFCs based on computational homogenization with SSRVEs. The SSRVEs were obtained by reducing the complexity of real microstructures based on statistical measures. Specifically, they were constructed by solving an optimization scheme which minimizes the difference between the power spectral density of target microstructures and that of the SSRVEs. As an extension to the method in^19^, we proposed to use a discretized parameterization in terms of integer quantities to improve the efficiency of the optimization sequence and thus, to be able to obtain a suitable SSRVE in reasonable time. The method was validated by performing representative numerical tests using simple target microstructures obtained by predefining values of a special selected parameterization. The validation results in Section 3 showed that the proposed method recover the predefined morphology more efficiently than the original method using the real-valued design variables.

In addition to this validation, an SSRVE has been constructed using a target microstructure obtained by X-ray computed tomography scans of a real DFC microstructure. It may be possible to construct an RVE from the target microstructure directly by choosing a subsection which matches the statistics of the available microstructure data. However, due to the high complexity of the real morphology, it is highly expensive to predict mechanical properties using such RVEs because a corresponding finite element model easily consists of more than 30 million elements. Therefore, the computing time of numerical material testing for DFCs can be significantly reduced using SSRVEs. In addition to the simple validation, it was shown that SSRVEs can indeed be applied to predict the macroscopic mechanical properties of real DFCs. By comparing the homogenized mechanical response of an SSRVE with experiments an accurate agreement was found.

The proposed method can be applied to design the production processes to optimize materials microstructures. In this paper, the SSRVE has been constructed from one target microstructure. For a mechanical simulation for injection molded components, several SSRVEs should be obtained from respective target microstructures representing point-to-point statistical variations due to injection molding process. However, thanks to the higher efficiency of the SSRVEs compared to classical RVEs such analysis will be enabled at a significantly reduced effort.
